# Expanding TNM for lung cancer through machine learning

**DOI:** 10.1111/1759-7714.13926

**Published:** 2021-03-13

**Authors:** Matthew Hueman, Huan Wang, Zhenqiu Liu, Donald Henson, Cuong Nguyen, Dean Park, Li Sheng, Dechang Chen

**Affiliations:** ^1^ Department of Surgical Oncology John P. Murtha Cancer Center, Walter Reed National Military Medical Center Bethesda Maryland USA; ^2^ Department of Biostatistics George Washington University Washington District of Columbia USA; ^3^ Department of Public Health Sciences Penn State Cancer Institute Hershey Pennsylvania USA; ^4^ Department of Preventive Medicine & Biostatistics, F. Edward Hébert School of Medicine Uniformed Services University of the Health Sciences Bethesda Maryland USA; ^5^ Department of Pathology Uniformed Services University of the Health Sciences Bethesda Maryland USA; ^6^ Department of Hematology‐Oncology John P. Murtha Cancer Center, Walter Reed National Military Medical Center Bethesda Maryland USA; ^7^ Department of Mathematics Drexel University Philadelphia Pennsylvania USA

**Keywords:** C‐index, lung cancer, machine learning, staging, survival

## Abstract

**Background:**

Expanding the tumor, lymph node, metastasis (TNM) staging system by accommodating new prognostic and predictive factors for cancer will improve patient stratification and survival prediction. Here, we introduce machine learning for incorporating additional prognostic factors into the conventional TNM for stratifying patients with lung cancer and evaluating survival.

**Methods:**

Data were extracted from SEER. A total of 77 953 patients were analyzed using factors including primary tumor (T), regional lymph node (N), distant metastasis (M), age, and histology type. Ensemble algorithm for clustering cancer data (EACCD) and C‐index were applied to generate prognostic groups and expand the current staging system.

**Results:**

With T, N, and M, EACCD stratified patients into 11 groups, resulting in a significantly higher accuracy in survival prediction than the 10 AJCC stages (C‐index = 0.7346 vs. 0.7247, increase in C‐index = 0.0099, 95% CI: 0.0091–0.0106, *p*‐value = 9.2 × 10^−147^). There nevertheless remained a strong association between the EACCD grouping and AJCC staging (rank correlation = 0.9289; *p*‐value = 6.7 × 10^−22^). A further analysis demonstrated that age and histological tumor could be integrated with the TNM. Data were stratified into 12 prognostic groups with an even higher prediction accuracy (C‐index = 0.7468 vs. 0.7247, increase in C‐index = 0.0221, 95% CI: 0.0212–0.0231, *p*‐value <5 × 10^−324^).

**Conclusions:**

EACCD can be successfully applied to integrate additional factors with T, N, M for lung cancer patients.

## Graphical abstract

Expanding the TNM by integrating additional factors/variables is challenging. This study used machine learning to create prognostic systems that expand the TNM. On the basis of primary tumor, regional lymph nodes, metastasis, age, and histology type, a prognostic system for lung cancer was created to improve the patient stratification and survival prediction of the TNM.

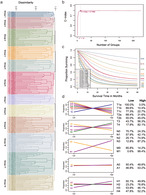

## INTRODUCTION

Cancer staging systems play an essential role in cancer medicine. They are used to develop prognosis, determine appropriate treatments, evaluate clinical trials, and convey clinical experiences. Lung cancer is classified according to the TNM staging system, based on anatomic factors of tumor extent, nodal status, and metastatic spread.^1^ The TNM provides basic information for tumor evaluation, treatment, and prognosis. However, lung cancer is no longer characterized by the anatomic extent of disease, but by a combination of various factors that can be clinical, biological, molecular, or genetic. Therefore, new and important factors need to be integrated in order to build prognostic systems that can improve evaluation and management decisions for lung cancer patients. Unfortunately, additional prognostic factors cannot be easily incorporated into the TNM staging system because the system is a result of consensus across many different areas.

Cox regression modeling^2^, ^3^ and tree modeling^4^, ^5^ are two major approaches that allow expansion of the TNM by integrating additional factors. Although Cox regression modeling can achieve a high accuracy in survival prediction, the risk groups extracted from the output (e.g., the nomogram) usually have a lower accuracy of survival prediction than the original model. Traditional survival tree modeling, which can be used to explicitly define prognostic groups, does not provide a high prediction accuracy in general.

In this study we describe a machine learning approach using the ensemble algorithm for clustering cancer data (EACCD)^6^, ^7^, ^8^, ^9^, ^10^, ^11^, ^12^, ^13^, ^14^, ^15^, ^16^, ^17^ to create prognostic systems for lung cancer. EACCD can adapt to any type and number of prognostic factors and generate systems that can be viewed as expansions of the TNM staging system. Variables/factors can be integrated by the EACCD to generate prognostic systems for refinements in patient stratification and outcome prediction that are needed for patient care such as monitoring of large scale therapeutic trials. We demonstrate the method by building two prognostic systems. One system, based on primary tumor (T), regional lymph node (N), and distant metastasis (M) was primarily employed to compare our approach with the AJCC. The second system, based on T, N, M, age (A), and histological type (H), expanded the traditional staging system based on T, N, M only. These prognostic systems from EACCD provide well‐defined patient stratification and high accuracy of survival prediction.

## METHODS

### Data source

Disease‐specific survival data with a primary diagnosis of lung cancer during 2010 to 2012 were obtained from 18 databases of the Surveillance, Epidemiology, and End Results Program (SEER) of the National Cancer Institute.^18^ This restriction on the year of diagnosis ensured a minimum five‐year follow‐up, since current release of SEER includes case reports up to the end of calendar year of 2017. As detailed below, patients diagnosed prior to 2010 were not included in our analysis. SEER cause‐specific death classification variable^19^ was used to capture all deaths related to lung cancer. Survival time was measured in months.

### Defining factors

SEER does not provide T, N, M categories in the eighth edition of the AJCC Cancer Staging Manual. Therefore, we used the derived AJCC seventh edition of the T, N, M variables^20^ to match the T, N, M levels for the eighth AJCC cancer staging systems. Specifically, with the SEER Collaborative Stage (CS) data collection system, we made the following reclassification of T: (1) classify seventh (edition) of T1a with CS tumor size^20^ ≤10 mm as eighth of T1a; (2) classify seventh of T1a with CS tumor size between 10 and 20 mm as 8th of T1b; (3) classify seventh of T1b as eighth of T1c; (4) classify seventh of T2a with CS tumor size between 30 and 40 mm as eighth of T2a; (5) classify seventh of T2a with CS tumor size between 40 and 50 mm as eighth of T2b; (6) classify as eighth of T3 (i) seventh of T2b and (ii) seventh of T3 with CS tumor size ≤70 mm; (7) classify as eighth of T4 (i) seventh of T3 with CS tumor size >70 mm and (ii) seventh of T4.

Because SEER started to include the derived AJCC‐7 T, N, M variables in 2010, patients with the year of diagnosis earlier than 2010 were not included in our analysis. This study investigated five factors: T, N, M, A, and H. Seven levels (T1a, T1b, T1c, T2a, T2b, T3, and T4) were used for T; four levels (N0, N1, N2, and N3) for N; and two levels (M0, M1) for M. Age and histological type were studied in this study since they are considered critical factors in survival prediction.^2^, ^3^, ^5^ Factor A had two levels: A0 (<70) and A1 (≥70). This cutoff, representing the lower boundary of senescence, was suggested by Gridelli et al.^21^ and was also used by Tanvetyanon et al.^2^ We note that 70 is also the median age for lung cancer in the SEER data. We studied four main histological types: squamous cell carcinoma (H1), small cell carcinoma (H2), adenocarcinoma (H3), and large cell carcinoma (H4). These four types were defined according to WHO histological classification of tumors of the lung.^22^ Table S1 lists the detailed definition of T, N, M, A, and H.

### Data management

**FIGURE 1:**
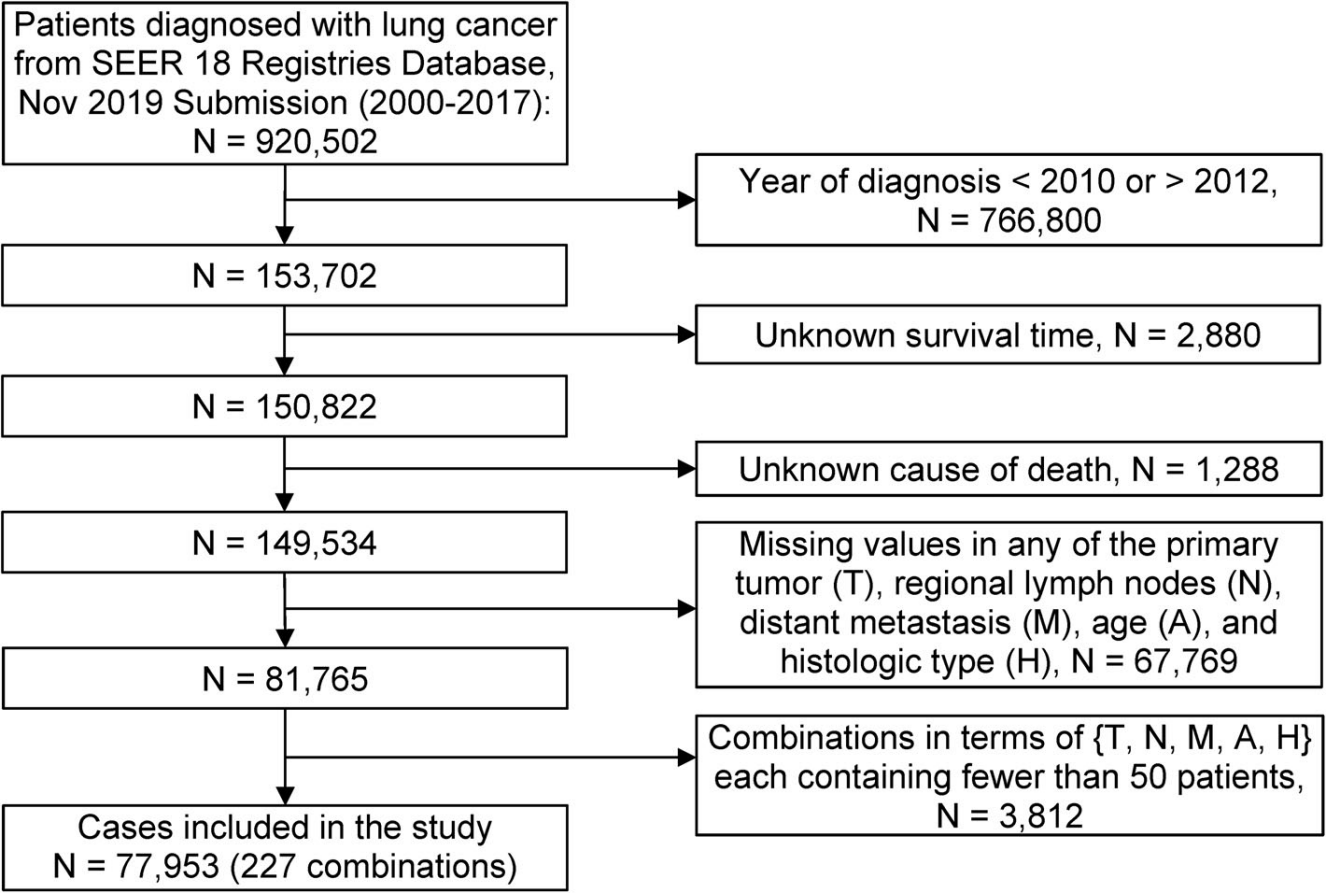
Clinical and demographic characteristics of the study cohort (*N* = 77 953)

Starting from the SEER lung cancer data with a primary diagnosis during 2010 to 2012, we selected all cases with complete information on the following factors/variables: T, N, M, A, H, survival time, and SEER cause‐specific death classification variable. Further selection of cases was made in terms of combinations of factors. We define a combination as a subset of the data corresponding to one level of each factor and we use levels of factors to denote combinations (e.g., T1N1M0A0H1 represents a subset of patients with T = T1, N = N1, M = M0, A = A0, H = H1). Due to the statistical techniques employed in EACCD, we required each combination to contain a sufficient number of patients in order to optimize robustness of results. We retained each combination of T, N, M, A, and H that contained a minimum of 50 cases. The resulting dataset contained 227 combinations of T, N, M, A, and H (77 953 cases, Figure 1 and Table 1). The median follow‐up of patients in the dataset was 70 months by the reverse Kaplan–Meier method.^23^

**TABLE 1:** Clinical and demographic characteristics of the study cohort (*N* = 77 953)

	N	%
Primary tumor		
T1a	1708	2.2
T1b	10 088	12.9
T1c	8830	11.3
T2a	7939	10.2
T2b	4789	6.1
T3	16 636	21.3
T4	27 963	35.9
Regional lymph node		
N0	34 422	44.2
N1	6253	8.0
N2	28 355	36.4
N3	8923	11.4
Distant metastasis		
M0	43 936	56.4
M1	34 017	43.6
Age		
A0	39 547	50.7
A1	38 406	49.3
Histological type		
H1	22 306	28.6
H2	10 449	13.4
H3	44 437	57.0
H4	761	1.0

### EACCD

The EACCD (Supplementary Appendix A) is a machine learning algorithm for clustering combinations. It first defines initial dissimilarities between two combinations, then obtains learned dissimilarities by an ensemble learning process, and then performs hierarchical clustering analysis to cluster combinations. The output of the algorithm is a tree‐structured dendrogram, showing the relationship among survival of patients in different combinations. Several approaches are available for each step. In this study, the initial dissimilarity between two combinations was defined by the Mann–Whitney parameter^24^ (Supplementary Appendix B); the ensemble learning process was based on the two‐phase Partitioning Around Medoids algorithm;^25^ and the minimax linkage method^26^ was chosen for hierarchical clustering. This is the first time the Mann–Whitney parameter and the minimax linkage have been used together.

### Prognostic systems

The dendrogram, obtained from the EACCD, can be cut horizontally to generate individual prognostic groups that serve the same role as the staging groups in the TNM. We cut the dendrogram in light of the C‐index.^27^ C‐index serves as an estimate of the probability that a subject who died at an earlier time had a shorter predicted survival time than a subject who died at a later time. Because of the tradeoff between model simplicity and prediction accuracy, we chose the “optimal” number of prognostic groups n* around the “knee” point of the C‐index curve (the C‐index vs. the number of prognostic groups).^12^, ^14^, ^15^, ^16^ Survival curves for the prognostic groups were plotted by using Kaplan–Meier estimates.^28^ The final prognostic system included the dendrogram, group assignment, C‐index, and survival curves for the prognostic groups.

## RESULTS

### Prognostic system for T, N, M

**FIGURE 2:**
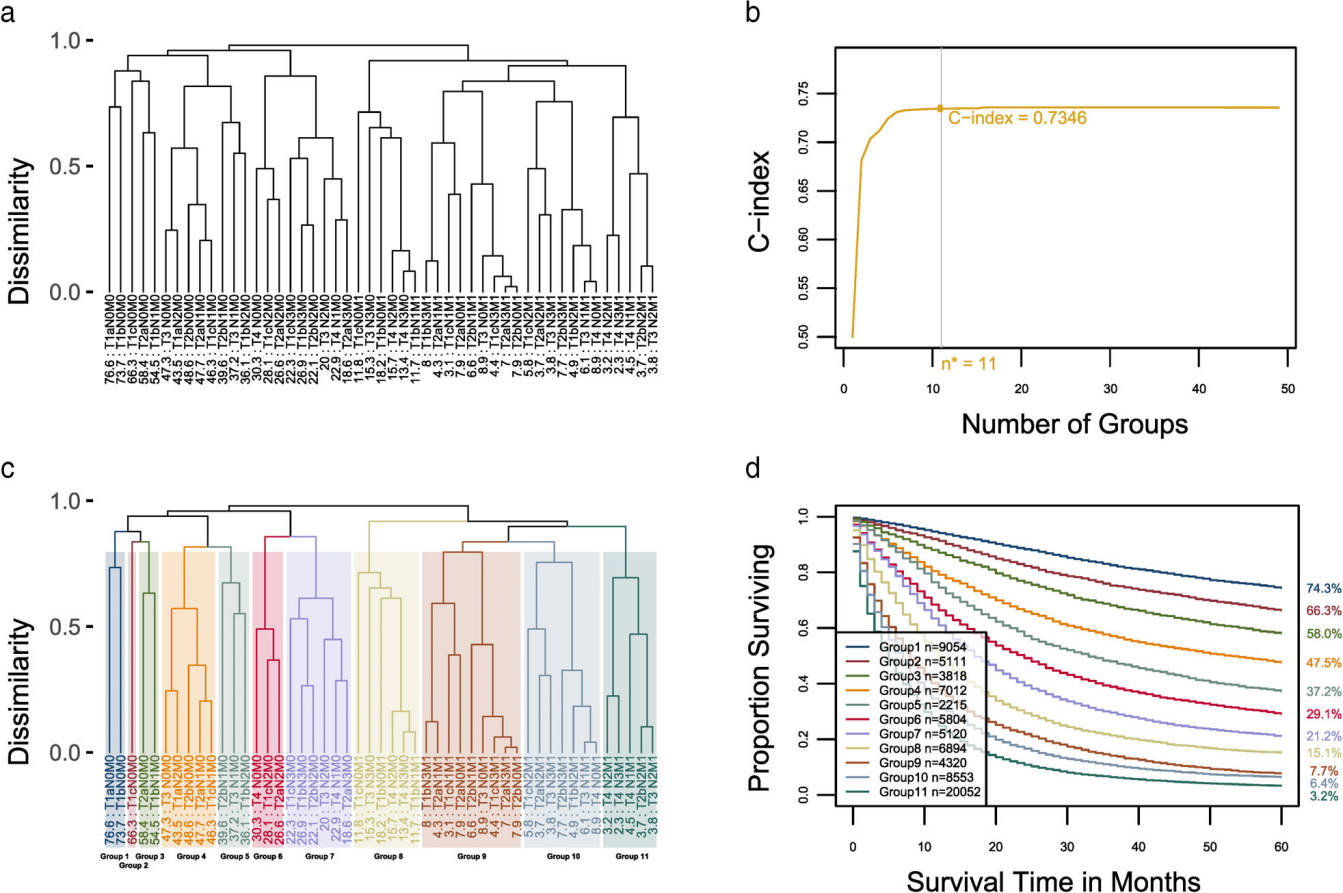
Creating ensemble algorithm for clustering cancer data (EACCD) prognostic groups on T, N, and M. (a) Dendrogram from running EACCD. A five‐year cancer‐specific survival rate in percentage is provided below each combination. (b) C‐index curve based on the dendrogram in panel (a). The knee point of the curve corresponds to 11 groups and a C‐index value of 0.7346. (c) Cutting the dendrogram in panel (a) according to n* = 11 suggested in panel (b) creates 11 prognostic groups. Group numbers are listed on the bottom of the dendrogram. (d) Lung cancer‐specific survival of 11 prognostic groups in panel (c). Five‐year cancer‐specific survival rates are listed on the right side

Applying the EACCD to the data based on T, N, and M yielded the dendrogram in Figure 2(a). The C‐index curve (Figure 2(b)) was used to find the optimal number of prognostic groups n*. The knee point of the curve corresponds to 11 groups (C‐index = 0.7346), which suggested n* = 11 (the C‐index slowly increases from 7 to 11 groups). Cutting the dendrogram into n* = 11 groups is shown in rectangles (Figure 2(c)). The survival curves for these 11 groups are seen in Figure 2(d). For convenience, the definition for all 11 groups is restated in the fourth column of Table S2. The resulting prognostic system for T, N, and M includes the dendrogram with cutting (Figure 2(c)), the groups in the fourth column of Table S2, and the survival curves (Figure 2(d)). This system contains 11 groups: group 1, group 2 … group 11 whose risk increases as the group number increases.

**FIGURE 3:**
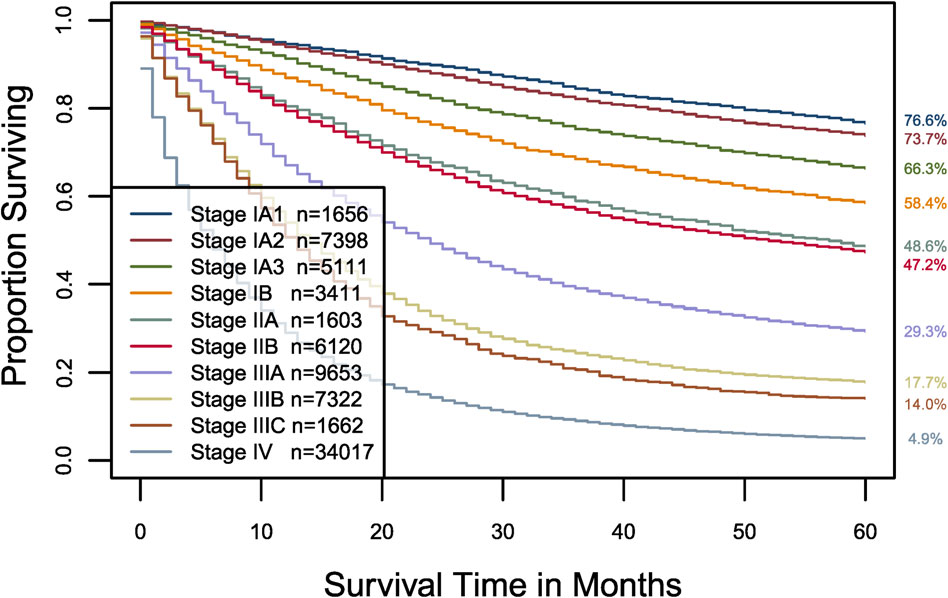
Cancer‐specific survival of AJCC stages defined in the fifth column in Table S2. The five‐year cancer‐specific survival rates for 10 stages are listed on the right side of the figure

For comparison, the eighth edition AJCC divides the data into 10 groups. Details are seen in the fifth column of Table S2 and Figure 3 (we did not include stage 0 and we treated stage M1a/b/c as M1). Calculation shows that the AJCC staging system has a C‐index of 0.7247. The *p*‐value of the C‐index based test^29^ for testing differences between the prediction accuracy of the above EACCD prognostic system (11 groups, C‐index = 0.7346) and the AJCC staging system TNM (10 groups, C‐index 0.7247) was 9.2 × 10^−147^. This shows that the EACCD system has a significantly higher survival prediction accuracy than the AJCC system.

### Prognostic system for T, N, M, A, and H

**FIGURE 4:**
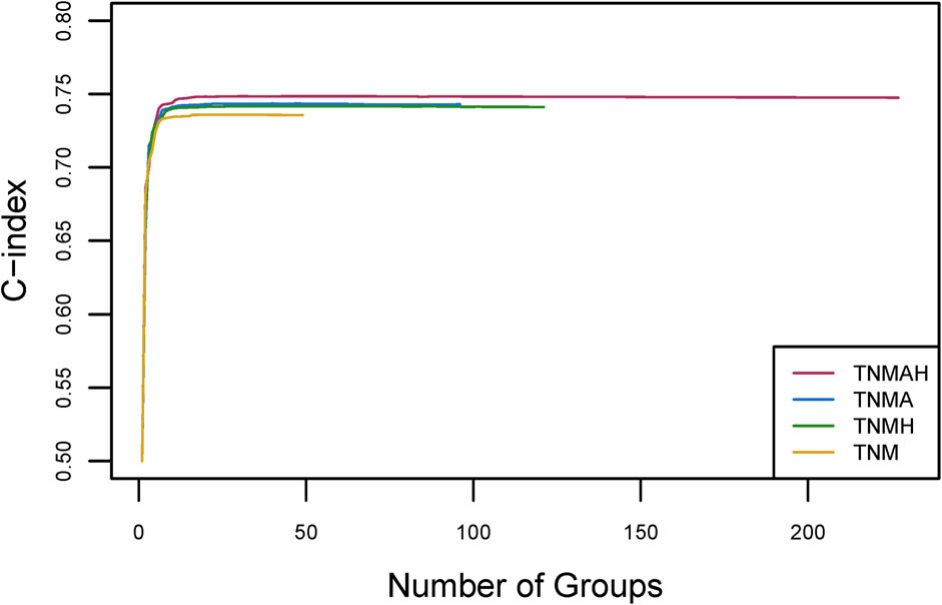
C‐index curves based on different factors

Before building the EACCD prognostic system for T, N, M, A, and H, we assessed the performance in survival prediction of the models for the following three sets of factors: {T, N, M, A}, {T, N, M, H}, and {T, N, M, A, H}, as compared with the model based on {T, N, M}. This is done by examining the C‐index curves for all these four scenarios (Figure 4). For more than three groups, the curves for {T, N, M, A} and {T, N, M, H} are higher than the curve for {T, N, M}. Therefore, adding A or H to {T, N, M} increases the C‐index and thus improves the prediction accuracy. The curve of {T, N, M, A, H} is the highest among all four curves, implying that adding both A and H to {T, N, M} leads to the biggest improvement on the prediction accuracy of {T, N, M}.

**FIGURE 5:**
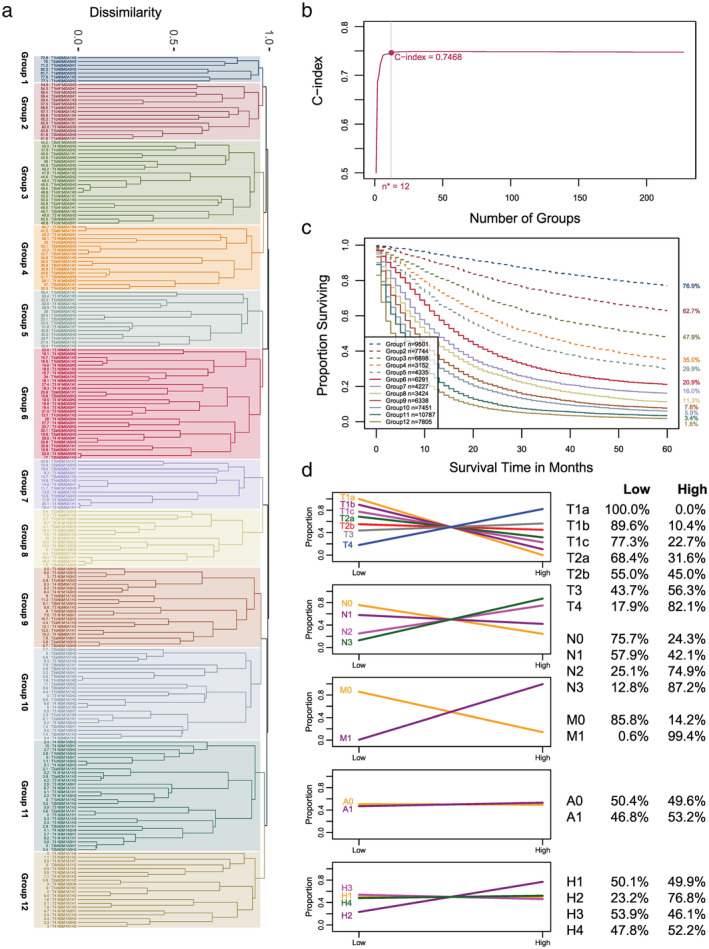
Ensemble algorithm for clustering cancer data (EACCD) prognostic groups on T, N, M, A, and H. (a) Dendrogram and cutting the dendrogram (shown in rectangles). A five‐year cancer‐specific survival rate in percentage is provided to the left of each combination. Cutting the dendrogram according to n* = 12 in panel (b) creates 12 prognostic groups. Listed on the left of the dendrogram are group numbers. (b) C‐index curve based on the dendrogram in panel (a). The knee point of the curve corresponds to 12 groups and a C‐index value of 0.7468. (c) Lung cancer‐specific survival of 12 prognostic groups in panel (a). The five‐year cancer‐specific survival rates for 12 groups are listed on the right side of the figure. (d) Distributions of patients over risk categories. In each row, one factor is concerned, and for each level of the factor, the distribution of patients (two proportions at two risk categories) is presented in two ways: Plot on the left and tabulation on the right

Applying the EACCD to the data based on T, N, M, A, and H yielded the dendrogram in Figure 5(a). The optimal number of prognostic groups n* = 12 with a corresponding C‐index of 0.7468 is indicated in Figure 5(b). Therefore, we cut the dendrogram into n* = 12 groups (rectangles in Figure 5(a)). Accordingly, the survival curves for the 12 prognostic groups can be plotted (Figure 5(c)). A detailed definition for all 12 groups is listed in Table S3.

## DISCUSSION

### Comparison with the TNM

The EACCD prognostic system based on TNM can be compared with the AJCC staging system in terms of both stratification and prediction. Previously, we showed that the EACCD system based on TNM (11 prognostic groups with C‐index = 0.7346) has a significantly higher survival prediction accuracy than the AJCC staging system (10 stage groups with C‐index = 0.7247). Below we compare the two systems by examining how patients are stratified.

In fact, there is a strong positive association between AJCC staging and EACCD grouping. Table 2 presents the distribution of patients of each of 10 AJCC stages over the 11 EACCD groups. The upper right and lower left corners of the table are filled with 0. Approximately, the higher stage the patient is assigned to by the AJCC system, the higher risk group the patient is assigned to by the EACCD, and vice versa. Indeed, the assignment to ordered stages and the assignment to ordered prognostic groups have a large Spearman's rank correlation coefficient^30^ of 0.9289 with a *p*‐value of 6.7 × 10^−22^.

**TABLE 2:** Contingency table between ensemble algorithm for clustering cancer data (EACCD) grouping and AJCC staging on the basis of T, N, M

AJCC\EACCD	1	2	3	4	5	6	7	8	9	10	11	Total
IA1	1656	0	0	0	0	0	0	0	0	0	0	1656
IA2	7398	0	0	0	0	0	0	0	0	0	0	7398
IA3	0	5111	0	0	0	0	0	0	0	0	0	5111
IB	0	0	3411	0	0	0	0	0	0	0	0	3411
IIA	0	0	0	1603	0	0	0	0	0	0	0	1603
IIB	0	0	407	5357	356	0	0	0	0	0	0	6120
IIIA	0	0	0	52	1859	5804	1938	0	0	0	0	9653
IIIB	0	0	0	0	0	0	3182	4140	0	0	0	7322
IIIC	0	0	0	0	0	0	0	1662	0	0	0	1662
IV	0	0	0	0	0	0	0	1092	4320	8553	20 052	34 017
Total	9054	5111	3818	7012	2215	5804	5120	6894	4320	8553	20 052	77 953

In summary, in predicting survival, the EACCD prognostic system on {T, N, M} has a significantly higher accuracy than the AJCC staging system TNM; in stratifying patients, the EACCD grouping and AJCC staging are strongly positively associated.

We note that the EACCD prognostic system on {T, N, M} can be further compared with the AJCC staging system TNM through validation datasets, preferably from sources other than SEER. For instance, when an appropriate validation set is available, EACCD and AJCC can be compared in terms of survival curves and values of C‐index on the validation set.

### Effect of factor levels on survival

The EACCD prognostic system on {T, N, M, A, H} allowed us to examine the effect of levels of individual factors on survival. To simplify the analysis, we considered the following two risk categories: low risk (groups 1–5) and high risk (groups 6–12) (this partition was suggested by the survival of prognostic groups shown in Figure 5(c). A graph is used to show how patients associated with a factor level are distributed across the two risk categories (Figure 5(d)).

The first row shows that as the T levels become more aggressive, patients are more likely to be classified into the high risk category.

The second and third rows reveal that patients with N0, or N1, or M0 status tend to have favorable survival while those with N2, or N3, or M1 have unfavorable survival.

The fourth row indicates that A0 and A1 curves are similar, both showing an approximately equal distribution in the two risk categories. This is the marginal effect of age, given the two risk categories. Earlier age was shown to be an important prognostic factor. These suggest that age should be considered in conjunction with other factors when informing prognosis.

The fifth row details the distribution of patients associated with each histological type. Patients with H1, H3, and H4 are approximately even distributed across the low and high risk categories, suggesting that squamous cell carcinoma, adenocarcinoma, and large cell carcinoma are not prone to high or low risk when these levels are presented alone. In comparison, small cell carcinoma shows a strong tendency towards high risk. (The H2 curve is increasing, with a small percentage of patients at low risk and a majority at high risk.) This finding reconfirms that small cell carcinoma and non‐small cell carcinoma play different roles in prognosis.^31^, ^32^

The above analysis shows how a factor level is associated with risk. Although these observations have been previously reported in the literature, this is the first time that these factor levels have been integrated together and explicitly highlighted in the ordered risk groups of the prognostic system TNMAH created in this study.

### Limitations of analyses

Cancer‐specific survival data were used in this study. Although the SEER cause‐specific death classification is determined by taking into account other elements (e.g., tumor sequence, site of the original cancer diagnosis, and comorbidities), death certificate errors can be problematic in estimating the cause‐specific survival. Another limitation is that the EACCD requires a relatively large size for each combination to produce robust estimates of survival. This report includes combinations with at least 50 cases. This may exclude some “rare” but interesting combinations. Improved estimates of survival can be achieved with a larger cutoff. Clearly, this requirement on the size of combinations will be met automatically when more data becomes available. Finally, due to the current restriction of SEER data, we derived the AJCC eighth edition of the T, N, M variables from those of the seventh edition, which could introduce some bias to this present study.

In conclusion, here we describe a machine learning approach based on EACCD and C‐index to refine the TNM system for lung cancer by integrating additional prognostic factors. We demonstrated the approach by using the SEER lung cancer data to create a prognostic system based on T, N, and M, which classifies patients in a way strongly positively correlated with the AJCC TNM staging system but has a higher accuracy for predicting survival. Using SEER, we created one computational prognostic system based on T, N, M, A, and H, which expanded (with additional factors) and improved (with a higher accuracy of survival prediction) the TNM for lung cancers. Results have shown that the machine learning approach takes into account both prediction and stratification and is analogous to the AJCC scheme in generating stages.

## CONFLICT OF INTEREST

The authors declare no conflict of interest.

## Supporting information


**Appendix S1**. Supporting Information
