# Supplementary material for: Expanding TNM for lung cancer through machine learning
Source: Thorac Cancer. 2021 Mar 13;12(9):1423–30. doi: 10.1111/1759-7714.13926 (PMC8088955; doi:10.1111/1759-7714.13926)
Supplement: Supplementary file 1 — Appendix S1. Supporting Information [file TCA-12-1423-s001.docx]

**Appendix A**

This appendix presents more details about the EACCD. The material is taken from the study by Hueman *et al*.^1^ and Yang *et al*.^2^

The EACCD^3^ is a machine learning algorithm designed to partition patients according to the survival time, censoring status as well as measurements on a sequence of selected categorical variables. Development has targeted its application and improvement.^1,2,4-12^ The algorithm consists of 3 steps: defining initial dissimilarities (in terms of the difference between survival functions) between combinations, computing learned dissimilarities, and performing hierarchical clustering of the combinations. Below is one version of the algorithm that utilizes the two-phase Partitioning Around Medoids algorithm (PAM).^13^

Given a collection of combinations $\{C_{1}, C_{2},\cdots,C_{n}\}$ and nonnegative weights $w_{1}, w_{2},..., w_{n}$ with $\sum_{k=1}^{n} w_{k}=1.$

1. Define the initial dissimilarity $dis₀(C_{i}, C_{j})$ for any pair $C_{i}$ and $C_{j}$.
2. For each $k$ with $1\leq k\leq n$, apply the two-phase PAM and the initial dissimilarities in Step 1 to partition combinations into $k$clusters, and define $\delta_{k}(i, j)=1$ if $C_{i}$ and $C_{j}$ are not assigned into the same cluster and $\delta_{k}(i, j)=0$ otherwise. Compute the learned dissimilarity $dis(C_{i}, C_{j})$ =$\sum_{k=1}^{n} w_{k}\delta_{k}(i, j)$.
3. Perform hierarchical clustering to cluster the combinations by using $dis(C_{i}, C_{j})$.

In Step 1, the initial dissimilarity can be defined as the value of a test statistic, such as the log-rank test statistic, Gehan-Wilcoxon test statistic, and Tarone-Ware test statistic. When the sizes of combinations are big, better initial dissimilarities can be defined by effect size based measures, such as hazard ratios and Mann-Whitney parameters.^8, 10, 11^

Step 2 utilizes initial dissimilarities in Step 1 and an ensemble process to compute the learned dissimilarities, which are more data driven than the initial dissimilarities. The two-phase PAM is used in the ensemble process to partition combinations. The results from PAM are then combined to generate the learned dissimilarity, which is simply the weighted percentage of the times two combinations are not placed into the same cluster by the PAM algorithm. One simple selection of weights is $w_{k}=1/kw$ with $w=1/1+1/2+...+1/n$ for $k=1, 2,..., n.$ In early versions of EACCD, learned dissimilarities were obtained by averaging the results from many runs of partition methods, which could take a long time to complete if a huge number of runs were used. In contrast, Step 2 above only requires to run PAM $n$ times, a number equal to the number of combinations.

Step 3 clusters the combinations by the learned dissimilarities from Step 2 and a linkage method. Single linkage, average linkage, complete linkage, minimax linkage, or other agglomerative hierarchical clustering methods may be used in this step.^14, 15^ The primary output of the algorithm is a dendrogram that provides a graphical summary of patients’ survival based on the levels of prognostic factors or variables.

In this paper, the initial dissimilarity in Step 1 is based on the Mann-Whitney parameter described below; the weights in Step 2 are chosen to be $w_{1}=...=w_{K}=1/n$; and the minimax linkage is used in Step 3.

**Appendix B**

The Mann-Whitney parameter originates from the widely used Mann-Whitney test.^16^ Let $T_{1}$ and $T_{2}$ denote the variables of survival time for patients from population 1 (with survival function $S_{1}(t)$) and population 2 (with survival function $S_{2}(t)$), respectively. The Mann-Whitney parameter is defined as the probability $P({T_{1}>T}_{2})$. The quantity $P({T_{1}>T}_{2})-0.5$ then suggests a difference between $S_{1}(t)$ and $S_{2}(t)$. Efron used $\hat{D}=-\int_{0}^{\infty} \hat{S}_{1}(t){d\hat{S}}_{2}(t)$to estimate $P({T_{1}>T}_{2})$ for censoring data, where $\hat{S}_{1}(t)$ and $\hat{S}_{2}(t)$ are Kaplan-Meier estimates of $S_{1}(t)$ and $S_{2}(t)$, respectively.^17^ Efron’s estimator requires that both $\hat{S}_{1}(t)$ and $\hat{S}_{2}(t)$ drop to 0 at the longest following-up time and is rather unstable when censoring occurs due to incomplete follow up.^18^ To overcome this problem, Wang proposed to use exponential tails to complete survival functions and then use the completed survival curves to estimate the Mann-Whitney parameter.^11^ More specifically, given two preselected positive constants $\tau_{1}$ and $\tau_{2}$, the survival functions $S_{1}(t)$ and $S_{2}(t)$ can be approximated by exponential functions beyond ${t=\tau}_{1}$ and ${t=\tau}_{2}$, respectively. Consequently, $P({T_{1}>T}_{2})$ is approximated by $D_{e}({\tau_{1},\tau}_{2})=A+B+C$, where $A=-\int_{0}^{min(\tau_{1},\tau_{2})} S_{1}(t) {dS}_{2}(t)$; $B=-\int_{\tau_{1}}^{\tau_{2}} e^{-\lambda_{1}t}{dS}_{2}(t)$ if $\tau_{1}<\tau_{2}$, $0$ if $\tau_{1}=\tau_{2}$, $\int_{\tau_{2}}^{\tau_{1}} S_{1}(t)\lambda_{2}e^{-\lambda_{2}t}dt$ if $\tau_{1}>\tau_{2}$; $C=\frac{\lambda_{2}}{\lambda_{1}+\lambda_{2}}e^{-(\lambda_{1}+\lambda_{2})max(\tau_{1},\tau_{2})}$; and $\lambda_{i}=-log(S_{i}(\tau_{i}))/\tau_{i}$ for $i=1, 2$. Accordingly, one can use the Kaplan-Meier estimates and Lebesgue-Stieltjes integration to compute $\hat{D}_{e}(\tau_{1},\tau_{2})=\hat{A}+\hat{B}+\hat{C}$, an estimate of $P({T_{1}>T}_{2})$. In this paper, $\tau_{1}$ and $\tau_{2}$ were set to be the maximum possible time by which the Kaplan-Meier estimates of the survival of all combinations can be calculated, and $|\hat{D}_{e}(\tau_{1},\tau_{2})-0.5|$ was used to compute the initial dissimilarity between two combinations.

1. Hueman M, Wang H, Henson D, Chen D. Expanding the TNM for cancers of the colon and rectum using machine learning: a demonstration. *ESMO Open.* 2019 Jun 1;4(3):e000518.
2. Yang C, Gardiner L, Wang H, Hueman M, Chen D. Creating prognostic systems for well differentiated thyroid cancer using machine learning. *Front Endocrinol.* 2019;10:288.
3. Chen D, Xing K, Henson D, Sheng L, Schwartz AM, Cheng X. Developing prognostic systems of cancer patients by ensemble clustering. *BioMed Res Int.* 2009 Jun 23;2009.
4. Wu D, Yang C, Wong S, Meyerle J, Zhang B, Chen D. An examination of TNM staging of melanoma by a machine learning algorithm. In 2012 International Conference on Computerized Healthcare (ICCH) 2012 Dec 17 (pp. 120-126). IEEE.
5. Qi R, Wu D, Sheng L, et al. On an ensemble algorithm for clustering cancer patient data. *BMC Syst Biol.* 2013 Oct;7(4):S9.
6. Chen D, Hueman MT, Henson DE, Schwartz AM. An algorithm for expanding the TNM staging system. *Future Oncol.* 2016 Apr;12(8):1015-24.
7. Chen D, Wang H, Sheng L, Hueman MT, Henson DE, Schwartz AM, Patel JA. An algorithm for creating prognostic systems for cancer. *J Med Syst.* 2016 Jul 1;40(7):160.
8. Wang H, Chen D, Hueman MT, Sheng L, Henson DE. Clustering big cancer data by effect sizes. In Proceedings of the Second IEEE/ACM International Conference on Connected Health: Applications, Systems and Engineering Technologies 2017 Jul 17 (pp. 58-63). IEEE.
9. Hueman MT, Wang H, Yang CQ, et al. Creating prognostic systems for cancer patients: A demonstration using breast cancer. *Cancer Med.* 2018 Aug;7(8):3611-21.
10. Wang H, Hueman M, Pan Q, et al. Creating Prognostic Systems by the Mann-Whitney Parameter. In 2018 IEEE/ACM International Conference on Connected Health: Applications, Systems and Engineering Technologies (CHASE) 2018 Sep 26 (pp. 33-39). IEEE.
11. Wang H. Development of Prognostic Systems for Cancer Patients. Doctoral dissertation, The George Washington University. 2020.
12. Praiss AM, Huang Y, Clair CM, Tergas AI, Melamed A, Khoury-Collado F, Hou JY, Hu J, Hur C, Hershman DL, Wright JD. Using machine learning to create prognostic systems for endometrial cancer. *Gynecol Oncol*. 2020 Dec 1;159(3):744-50.
13. Kaufman L, Rousseeuw PJ. Finding groups in data: an introduction to cluster analysis. Hoboken, New Jersey: John Wiley & Sons; 1990.
14. Hastie T, Tibshirani R, Friedman J. The elements of statistical learning: prediction, inference and data mining: Springer-Verlag, New York, 2009.
15. Bien J, Tibshirani R. Hierarchical clustering with prototypes via minimax linkage. *J Am Stat Assoc.* 2011 Sep 1;106(495):1075-84.
16. Mann HB, Whitney DR. On a test of whether one of two random variables is stochastically larger than the other. *Ann Math Stat.* (1947) 18:50-60
17. Efron B. The two sample problem with censored data. In M. Lucien, Le Cam and Jerzy N (eds) Fifth Berkeley Symposium on Mathematical Statistics and Probability, Statistical Laboratory of the University of California, Berkeley, June 21–18 July 1965 and 27 December 1965–7 January 1966, p.666. Berkeley, Calif: University of California Press. (1967)
18. Brentnall AR, Cuzick J. Use of the concordance index for predictors of censored survival data. *Stat Methods Med Res.* (2018) 27(8): 2359-73

**Table S1** Definitions of levels of T, N, M, A, and H for SEER lung cancer patients. Refer to AJCC Cancer Staging Manual^1^, SEER Research Data Record Description^2^, and WHO Classification of Tumours of the Lung, Pleura, Thymus and Heart^3^ for specifics of the 3rd column.

| ***Prognostic Factors*** | ***Levels*** | ***Definitions*** |
| --- | --- | --- |
| *Primary Tumor (T)* | *T1* | *Tumor ≤3 cm in greatest dimension, surrounded by lung or visceral pleura, without*  *bronchoscopic evidence of invasion more proximal than the lobar bronchus (i.e., not in*  *the main bronchus)* |
|  | *T1a* | *Tumor ≤1 cm in greatest dimension. A superficial, spreading tumor of any size whose invasive component is limited to the bronchial wall and may extend proximal to the main bronchus also is classified as T1a, but these tumors are uncommon.* |
|  | *T1b* | *Tumor >1 cm but ≤2 cm in greatest dimension* |
|  | *T1c* | *Tumor >2 cm but ≤3 cm in greatest dimension* |
|  | *T2* | *Tumor >3 cm but ≤5 cm or having any of the following features:*   - *Involves the main bronchus regardless of distance to the carina, but without involvement of the carina* - *Invades visceral pleura (PL1 or PL2)* - *Associated with atelectasis or obstructive pneumonitis that extends to the hilar region, involving part or all of the lung*   *T2 tumors with these features are classified as T2a if ≤4 cm or if the size cannot be determined and T2b if >4 cm but ≤5 cm.* |
|  | *T2a* | *Tumor >3 cm but ≤4 cm in greatest dimension* |
|  | *T2b* | *Tumor >4 cm but ≤5 cm in greatest dimension* |
|  | *T3* | *Tumor >5 cm but ≤7 cm in greatest dimension or directly invading any of the following: parietal pleura (PL3), chest wall (including superior sulcus tumors), phrenic nerve, parietal pericardium; or separate tumor nodule(s) in the same lobe as the primary* |
|  | *T4* | *Tumor >7 cm or tumor of any size invading one or more of the following: diaphragm, mediastinum, heart, great vessels, trachea, recurrent laryngeal nerve, esophagus, vertebral body, or carina; separate tumor nodule(s) in an ipsilateral lobe different from that of the primary* |
| *Regional Lymph Node (N)* | *N0* | *No regional lymph node metastasis* |
|  | *N1* | *Metastasis in ipsilateral peribronchial and/or ipsilateral hilar lymph nodes and intrapulmonary nodes, including involvement by direct extension* |
|  | *N2* | *Metastasis in ipsilateral mediastinal and/or subcarinal lymph node(s)* |
|  | *N3* | *Metastasis in contralateral mediastinal, contralateral hilar, ipsilateral or contralateral scalene, or supraclavicular lymph node(s)* |
| *Distant Metastasis (M)* | *M0* | *No distant metastasis* |
|  | *M1* | *Distant metastasis* |
| *Age (A)* | *A0* | *Age < 70* |
|  | *A1* | *Age ≥ 70* |
| *Histologic Type (H)* | *H1* | *Squamous cell carcinoma (ICD-O-3 = 8070, 8052, 8084, 8073, 8083)* |
|  | *H2* | *Small cell carcinoma (ICD-O-3 = 8041, 8045)* |
|  | *H3* | *Adenocarcinoma (ICD-O-3 = 8140, 8255, 8550, 8260, 8250, 8252, 8253, 8254, 8230, 8333, 8480, 8470, 8490, 8310)* |
|  | *H4* | *Large cell carcinoma (ICD-O-3 = 8012, 8013, 8123, 8082, 8014)* |

1. Amin MB, Edge S, Greene F, Byrd DR, Brookland RK, Washington MK, et al. AJCC cancer staging manual. 8th ed. Springer International Publishing; 2017.
2. SEER Research Data Record Description. 2020.[https://seer.cancer.gov/data-software/documentation/seerstat/nov2019/TextData.FileDescription.pdf](https://seer.cancer.gov/data-software/documentation/seerstat/nov2018/TextData.FileDescription.pdf) Accessed 27 Dec 2020.
3. Travis WD, Brambilla E, Müller-Hermelink HK, Harris CC, editors. Pathology and Genetics of Tumours of the Lung, Pleura, Thymus and Heart. World Health Organization Classification of Tumours, Lyon: IARC Press; 2004.

**Table S2** EACCD and AJCC grouping of lung cancer patients according to T, N, and M.

| **T** | **N** | **M** | **EACCD**  **prognostic group** | **AJCC**  **Staging group** |
| --- | --- | --- | --- | --- |
| T1a | N0 | M0 | 1 | IA1 |
| T1b | N0 | M0 | 1 | IA2 |
| T1c | N0 | M0 | 2 | IA3 |
| T1b | N1 | M0 | 3 | IIB |
| T2a | N0 | M0 | 3 | IB |
| T1a | N2 | M0 | 4 | IIIA |
| T1c | N1 | M0 | 4 | IIB |
| T2a | N1 | M0 | 4 | IIB |
| T2b | N0 | M0 | 4 | IIA |
| T3 | N0 | M0 | 4 | IIB |
| T1b | N2 | M0 | 5 | IIIA |
| T2b | N1 | M0 | 5 | IIB |
| T3 | N1 | M0 | 5 | IIIA |
| T1c | N2 | M0 | 6 | IIIA |
| T2a | N2 | M0 | 6 | IIIA |
| T4 | N0 | M0 | 6 | IIIA |
| T1b | N3 | M0 | 7 | IIIB |
| T1c | N3 | M0 | 7 | IIIB |
| T2a | N3 | M0 | 7 | IIIB |
| T2b | N2 | M0 | 7 | IIIA |
| T3 | N2 | M0 | 7 | IIIB |
| T4 | N1 | M0 | 7 | IIIA |
| T1b | N0 | M1 | 8 | IV |
| T1b | N1 | M1 | 8 | IV |
| T1c | N0 | M1 | 8 | IV |
| T3 | N3 | M0 | 8 | IIIC |
| T4 | N2 | M0 | 8 | IIIB |
| T4 | N3 | M0 | 8 | IIIC |
| T1b | N3 | M1 | 9 | IV |
| T1c | N1 | M1 | 9 | IV |
| T1c | N3 | M1 | 9 | IV |
| T2a | N0 | M1 | 9 | IV |
| T2a | N1 | M1 | 9 | IV |
| T2a | N3 | M1 | 9 | IV |
| T2b | N0 | M1 | 9 | IV |
| T2b | N1 | M1 | 9 | IV |
| T3 | N0 | M1 | 9 | IV |
| T1b | N2 | M1 | 10 | IV |
| T1c | N2 | M1 | 10 | IV |
| T2a | N2 | M1 | 10 | IV |
| T2b | N3 | M1 | 10 | IV |
| T3 | N1 | M1 | 10 | IV |
| T3 | N3 | M1 | 10 | IV |
| T4 | N0 | M1 | 10 | IV |
| T2b | N2 | M1 | 11 | IV |
| T3 | N2 | M1 | 11 | IV |
| T4 | N1 | M1 | 11 | IV |
| T4 | N2 | M1 | 11 | IV |
| T4 | N3 | M1 | 11 | IV |

**Table S3** EACCD grouping of lung cancer patients according to T, N, M, A, and H.

| **T** | **N** | **M** | **A** | **H** |
| --- | --- | --- | --- | --- |
| **Group 1** | | | | |
| T1a | N0 | M0 | A0 | H3 |
| T1a | N0 | M0 | A1 | H3 |
| T1b | N0 | M0 | A0 | H1 |
| T1b | N0 | M0 | A0 | H3 |
| T1b | N0 | M0 | A1 | H3 |
| T1c | N0 | M0 | A0 | H3 |
| T2a | N0 | M0 | A0 | H3 |
| **Group 2** | | | | |
| T1a | N0 | M0 | A0 | H1 |
| T1a | N0 | M0 | A1 | H1 |
| T1b | N0 | M0 | A0 | H4 |
| T1b | N0 | M0 | A1 | H1 |
| T1b | N0 | M0 | A1 | H4 |
| T1b | N1 | M0 | A0 | H1 |
| T1b | N1 | M0 | A0 | H3 |
| T1c | N0 | M0 | A0 | H1 |
| T1c | N0 | M0 | A1 | H3 |
| T1c | N1 | M0 | A0 | H3 |
| T2a | N0 | M0 | A0 | H1 |
| T2a | N0 | M0 | A1 | H3 |
| T2a | N1 | M0 | A0 | H3 |
| T2b | N0 | M0 | A0 | H3 |
| T3 | N0 | M0 | A0 | H3 |
| **Group 3** | | | | |
| T1a | N2 | M0 | A0 | H3 |
| T1b | N0 | M0 | A0 | H2 |
| T1b | N1 | M0 | A1 | H1 |
| T1b | N1 | M0 | A1 | H3 |
| T1b | N2 | M0 | A0 | H1 |
| T1b | N2 | M0 | A0 | H3 |
| T1c | N0 | M0 | A0 | H2 |
| T1c | N0 | M0 | A1 | H1 |
| T1c | N1 | M0 | A0 | H1 |
| T1c | N1 | M0 | A1 | H3 |
| T2a | N0 | M0 | A0 | H2 |
| T2a | N0 | M0 | A1 | H1 |
| T2a | N1 | M0 | A0 | H1 |
| T2b | N0 | M0 | A0 | H1 |
| T2b | N0 | M0 | A1 | H3 |
| T2b | N1 | M0 | A0 | H1 |
| T2b | N1 | M0 | A0 | H3 |
| T3 | N0 | M0 | A0 | H1 |
| T3 | N0 | M0 | A1 | H3 |
| T3 | N1 | M0 | A0 | H3 |
| T4 | N0 | M0 | A0 | H3 |
| T4 | N0 | M0 | A0 | H4 |
| **Group 4** | | | | |
| T1b | N0 | M0 | A1 | H2 |
| T1b | N2 | M0 | A1 | H3 |
| T1c | N0 | M0 | A1 | H2 |
| T1c | N2 | M0 | A0 | H3 |
| T2a | N1 | M0 | A1 | H1 |
| T2a | N1 | M0 | A1 | H3 |
| T2a | N2 | M0 | A0 | H1 |
| T2a | N2 | M0 | A0 | H3 |
| T2b | N0 | M0 | A1 | H1 |
| T2b | N1 | M0 | A1 | H1 |
| T2b | N1 | M0 | A1 | H3 |
| T2b | N2 | M0 | A0 | H3 |
| T3 | N0 | M0 | A0 | H2 |
| T3 | N0 | M0 | A0 | H4 |
| T3 | N0 | M0 | A1 | H4 |
| T3 | N1 | M0 | A0 | H1 |
| T4 | N1 | M0 | A0 | H3 |
| **Group 5** | | | | |
| T1b | N2 | M0 | A0 | H2 |
| T1b | N2 | M0 | A1 | H1 |
| T1b | N3 | M0 | A0 | H3 |
| T1c | N1 | M0 | A1 | H1 |
| T1c | N2 | M0 | A0 | H1 |
| T1c | N2 | M0 | A0 | H2 |
| T1c | N2 | M0 | A1 | H3 |
| T1c | N3 | M0 | A0 | H3 |
| T2a | N0 | M0 | A1 | H2 |
| T2a | N2 | M0 | A1 | H3 |
| T2b | N2 | M0 | A0 | H1 |
| T3 | N0 | M0 | A1 | H1 |
| T3 | N1 | M0 | A1 | H3 |
| T3 | N2 | M0 | A0 | H3 |
| T4 | N0 | M0 | A1 | H3 |
| **Group 6** | | | | |
| T1b | N0 | M1 | A0 | H3 |
| T1b | N2 | M0 | A1 | H2 |
| T1b | N3 | M0 | A1 | H3 |
| T1c | N2 | M0 | A1 | H1 |
| T1c | N3 | M0 | A1 | H3 |
| T2a | N2 | M0 | A0 | H2 |
| T2a | N2 | M0 | A1 | H1 |
| T2a | N3 | M0 | A0 | H3 |
| T2a | N3 | M0 | A1 | H1 |
| T2a | N3 | M0 | A1 | H3 |
| T2b | N2 | M0 | A0 | H2 |
| T2b | N2 | M0 | A1 | H3 |
| T3 | N0 | M0 | A1 | H2 |
| T3 | N1 | M0 | A1 | H1 |
| T3 | N2 | M0 | A0 | H1 |
| T3 | N2 | M0 | A0 | H2 |
| T3 | N2 | M0 | A1 | H3 |
| T3 | N3 | M0 | A0 | H2 |
| T3 | N3 | M0 | A0 | H3 |
| T4 | N0 | M0 | A0 | H1 |
| T4 | N0 | M0 | A0 | H2 |
| T4 | N1 | M0 | A0 | H1 |
| T4 | N1 | M0 | A0 | H2 |
| T4 | N1 | M0 | A1 | H3 |
| T4 | N2 | M0 | A0 | H2 |
| T4 | N2 | M0 | A0 | H3 |
| T4 | N2 | M0 | A0 | H4 |
| T4 | N3 | M0 | A0 | H2 |
| T4 | N3 | M0 | A0 | H3 |
| **Group 7** | | | | |
| T1b | N0 | M1 | A1 | H1 |
| T1b | N1 | M1 | A0 | H3 |
| T1c | N0 | M1 | A0 | H3 |
| T1c | N2 | M0 | A1 | H2 |
| T2a | N0 | M1 | A0 | H3 |
| T2b | N2 | M0 | A1 | H1 |
| T2b | N2 | M0 | A1 | H2 |
| T3 | N2 | M0 | A1 | H1 |
| T3 | N3 | M0 | A0 | H1 |
| T4 | N0 | M0 | A1 | H1 |
| T4 | N2 | M0 | A0 | H1 |
| T4 | N2 | M0 | A1 | H3 |
| T4 | N3 | M0 | A0 | H1 |
| **Group 8** | | | | |
| T1b | N3 | M1 | A0 | H3 |
| T1c | N1 | M1 | A0 | H3 |
| T2a | N1 | M1 | A0 | H3 |
| T2a | N2 | M0 | A1 | H2 |
| T2a | N3 | M1 | A0 | H3 |
| T2b | N0 | M1 | A0 | H3 |
| T3 | N0 | M1 | A0 | H1 |
| T3 | N0 | M1 | A0 | H3 |
| T3 | N1 | M1 | A0 | H3 |
| T3 | N3 | M0 | A1 | H1 |
| T3 | N3 | M0 | A1 | H3 |
| T4 | N0 | M1 | A0 | H3 |
| T4 | N1 | M0 | A1 | H1 |
| T4 | N3 | M0 | A1 | H1 |
| T4 | N3 | M0 | A1 | H3 |
| **Group 9** | | | | |
| T1b | N0 | M1 | A1 | H3 |
| T1b | N2 | M1 | A0 | H3 |
| T1c | N0 | M1 | A1 | H1 |
| T1c | N0 | M1 | A1 | H3 |
| T1c | N2 | M1 | A0 | H3 |
| T1c | N3 | M1 | A0 | H3 |
| T2a | N0 | M1 | A0 | H1 |
| T2a | N1 | M1 | A1 | H3 |
| T2a | N2 | M1 | A0 | H3 |
| T2b | N1 | M1 | A0 | H3 |
| T2b | N2 | M1 | A0 | H3 |
| T3 | N0 | M1 | A0 | H2 |
| T3 | N1 | M1 | A0 | H1 |
| T3 | N2 | M0 | A1 | H2 |
| T3 | N2 | M1 | A0 | H3 |
| T3 | N3 | M1 | A0 | H2 |
| T3 | N3 | M1 | A0 | H3 |
| T4 | N0 | M0 | A1 | H2 |
| T4 | N1 | M1 | A0 | H3 |
| T4 | N2 | M0 | A1 | H1 |
| T4 | N2 | M0 | A1 | H2 |
| **Group 10** | | | | |
| T1b | N2 | M1 | A1 | H3 |
| T1b | N3 | M1 | A1 | H3 |
| T1c | N1 | M1 | A1 | H3 |
| T1c | N2 | M1 | A1 | H3 |
| T1c | N3 | M1 | A1 | H3 |
| T2a | N0 | M1 | A1 | H1 |
| T2a | N0 | M1 | A1 | H3 |
| T2a | N2 | M1 | A0 | H1 |
| T2a | N2 | M1 | A0 | H2 |
| T2a | N3 | M1 | A0 | H2 |
| T2b | N0 | M1 | A0 | H1 |
| T2b | N0 | M1 | A1 | H1 |
| T2b | N0 | M1 | A1 | H3 |
| T2b | N1 | M1 | A1 | H3 |
| T2b | N3 | M1 | A0 | H3 |
| T2b | N3 | M1 | A1 | H3 |
| T3 | N0 | M1 | A1 | H3 |
| T3 | N1 | M1 | A0 | H2 |
| T3 | N2 | M1 | A0 | H2 |
| T3 | N3 | M1 | A0 | H1 |
| T4 | N0 | M1 | A0 | H2 |
| T4 | N0 | M1 | A1 | H3 |
| T4 | N2 | M1 | A0 | H3 |
| T4 | N3 | M0 | A1 | H2 |
| **Group 11** | | | | |
| T1b | N2 | M1 | A0 | H2 |
| T1b | N2 | M1 | A1 | H2 |
| T1c | N2 | M1 | A0 | H1 |
| T1c | N2 | M1 | A0 | H2 |
| T2a | N2 | M1 | A1 | H1 |
| T2a | N2 | M1 | A1 | H3 |
| T2a | N3 | M1 | A1 | H3 |
| T2b | N2 | M1 | A0 | H1 |
| T2b | N2 | M1 | A0 | H2 |
| T2b | N2 | M1 | A1 | H1 |
| T2b | N2 | M1 | A1 | H3 |
| T3 | N0 | M1 | A1 | H1 |
| T3 | N0 | M1 | A1 | H2 |
| T3 | N1 | M1 | A1 | H1 |
| T3 | N1 | M1 | A1 | H3 |
| T3 | N2 | M1 | A0 | H1 |
| T3 | N2 | M1 | A1 | H3 |
| T3 | N3 | M1 | A1 | H1 |
| T3 | N3 | M1 | A1 | H3 |
| T4 | N0 | M1 | A0 | H1 |
| T4 | N0 | M1 | A1 | H1 |
| T4 | N1 | M1 | A0 | H2 |
| T4 | N1 | M1 | A1 | H3 |
| T4 | N2 | M1 | A0 | H1 |
| T4 | N2 | M1 | A0 | H2 |
| T4 | N2 | M1 | A0 | H4 |
| T4 | N3 | M1 | A0 | H1 |
| T4 | N3 | M1 | A0 | H2 |
| T4 | N3 | M1 | A0 | H3 |
| **Group 12** | | | | |
| T1c | N2 | M1 | A1 | H2 |
| T2a | N2 | M1 | A1 | H2 |
| T2b | N2 | M1 | A1 | H2 |
| T3 | N2 | M1 | A0 | H4 |
| T3 | N2 | M1 | A1 | H1 |
| T3 | N2 | M1 | A1 | H2 |
| T3 | N3 | M1 | A1 | H2 |
| T4 | N0 | M1 | A0 | H4 |
| T4 | N0 | M1 | A1 | H2 |
| T4 | N1 | M1 | A0 | H1 |
| T4 | N1 | M1 | A1 | H1 |
| T4 | N1 | M1 | A1 | H2 |
| T4 | N2 | M1 | A1 | H1 |
| T4 | N2 | M1 | A1 | H2 |
| T4 | N2 | M1 | A1 | H3 |
| T4 | N2 | M1 | A1 | H4 |
| T4 | N3 | M1 | A0 | H4 |
| T4 | N3 | M1 | A1 | H1 |
| T4 | N3 | M1 | A1 | H2 |
| T4 | N3 | M1 | A1 | H3 |
